# Notes and News

**Published:** 1890-01-25

**Authors:** 


### Notes and News.

Collected and Communicated.

A conference of medical men and hospital administrators in Birmingham met in that town on January 15, to consider whether the hospitals there are abused, and if so, what remedy can be applied. In the course of the discussion which took place, it was stated that though the enquiry into the matter of the giving of public relief in the Birmingham ordinary hospitals, in the case of the Charity Organisation Society only showed one unsatisfactory case out of over 250 applications, yet in an enquiry made at the Royal Free Hospital it was found that out of 641 cases only 169 were suitable for free medical relief. Of the others 12 were able to afford to pay a private practitioner; 231 to subscribe to provident dispensaries; while 57 should have been relieved by the Poor Law; in 103 cases false addresses were given; and in 69 sufficient information could not be obtained. From Mr. Priestley Smith's remarks, it would appear that the cause of the defects in the present management of hospitals in Birmingham arose from the number competing for public support, there being no less than thirteen voluntary hospitals in the town, and this competitive system led them into attempting and professing more than they were able to do, being unjust to those who supported, injurious to those who used the hospitals, and an imposition on the medical profession. They were always begging for money, but with all their large expenditure they did not treat their patients properly, out-patients being kept waiting for hours, and then hurried over, while in-patients were more numerous than the beds at their disposal.

As a result of the discussion, a committee, the constitution of which was left to the Mayor of Birmingham, was appointed to enquire "into the question of the medical treatment of the poorer classes," and to report with suggestions as to any desirable reform to a future meeting of the conference.

Dr. Lanceraux has been speaking before the Paris Academy of Medicine, on the prevalence of phthisis in cities and its absence amongst people leading the lives of savages. He also said that the abuse of spirituous liquors brought on consumption. In that disease it was more necessary to combat circumstances than to seek for the microbe. It was, he affirmed, requisite to make the public powers understand that healthy air was quite as much as, if not more than, pure water an indispensable element to the health of man. He urged that pure water was the best safeguard against typhus fever, and predicted that the ravages of consumption would be as effectually combated by pure air as those of typhus were by pure water.

At Northwich Victoria Infirmary Christmas was kept in the orthodox manner; the usual roast beef, evergreens, and presents marking the festive season. The farce "All a Hoax" was acted by some of the friends of the hospital, and a concert was given in which the patients assisted. The matron, Miss Davies, was thanked for her hospitality.

Dr. Spottiswoode Cameron, medical officer for Leeds, has put before the Sanitary Committee a report concerning the notification of infectious diseases and the hospital accommodation for the same. Dr. Cameron wrote to fifty-six towns which have adopted the Infectious Diseases Act, and found that, where adopted, increased hospital accommodation was almost always necessary. Blackburn is borrowing money to build one, Sunderland is just furnishing one, and of six towns who have not yet established a hospital, the medical officer of Nelson has recommended his board to erect one. Dr. Cameron concludes that it is useless to adopt the Act unless you have an infectious hospital. If anyone is calculated to give an opinion it is Dr. Spottiswoode Cameron, for he is the author of the admirable little handbook, "Hints as to the Working of the Infectious Disease (Notification) Act, 1889."

At the weekly meeting of the London County Council, held at the Guildhall on Tuesday, the Earl of Rosebery, chairman, presiding, Mr. P. M. Martineau brought up a report of the Asylums Committee, stating that they had had under consideration the construction of an operating-room at Banstead Asylum. At present there was no place for minor operations, excepting the dispensary, which was small and ill-adapted for the purpose; and for major operations, which were not infrequent, the day-room of one of the infirmaries had to be used, necessitating the temporary removal of all the patients. The committee were informed that an operating-room had for some time past been under consideration at the instance of the Commissioners in Lunacy, who, in the report on their last inspection of the asylum, strongly urged the necessity for such a room. In all the circumstances the committee recommended "That, subject to an estimate being submitted to the council by the Finance Committee as required by the statute, the committee be authorised to erect an operating-room at the Banstead Asylum, at an estimated cost of £500." The report was adopted. Can anything be more foolish than to acknowledge the necessity for an operating-room and yet to refuse to have trained nurses in this asylum, where there are often twenty deaths a month?

The first annual report of Bournemouth Royal Victoria Hospital is specially interesting just now as prominence is given to that institution by the Prince of Wales's visit to its wards. Since the hospital has been opened about twenty beds on an average have been occupied, which fact shows the urgent necessity there was for increased accommodation beyond the seven beds at the Cottage Hospital. The Eastern Dispensary has been found most useful by the poor living at the eastern end of the district, and the work there is likely to increase. Twelve lady visitors were appointed in October, under the rules of the institution, to read to and otherwise interest the patients. They have also undertaken the management of the Samaritan fund started at the instance of the Dowager Lady Gibson Craig. The intention of this fund is to assist the families of those who are left in want, owing to the breadwinner being removed to the hospital, and to give a helping hand to patients leaving the hospital. The receipts have amounted to £1,266 5s. 8d. This includes £49 9s. 9d. from the dispensary, and three life governors' donations of £26 5s. each. It is evident that the subscription list must be considerably enlarged if the establishment is to be maintained in a state of efficiency. It is a matter of surprise that the income this year is very little larger than that of the old Cottage Hospital, whose receipts in 1888 were about £1,000.

The following vacancies are announced this week, the amount of the salary in each case being given after the name of the institution :

**Resident Medical Officers.**

Fisherton Asylum. Assistant Medical Officer — £100, board, &c.
Birmingham General Hospital. Two Assistant House Surgeons—Board, &c.
Leeds Infirmary. Medical Officer and Pathologist—£100, board, &c.
Liverpool Stanley Hospital. Junior House Surgeon—£70, board, &c.

**Non-Resident Medical Officers.**

Parochial Board of Bersay and Harray. Medical Officer.
Sowerby Bridge Local Board of Health. Medical Officer—£30.
Chelsea Hospital for Women. Anæsthetist.
Borough of Bradford. Public Analyst.

---

The Queen has been graciously pleased to forward five brace of pheasants and 100lb. of cast linen to the Great Northern Central Hospital, Holloway-road.

---

The New York Hospital has been lighted throughout by electricity, and physicians, patients, and management are equally delighted with the result. Sanitary motives dictated the change, as a more even temperature could be kept in the wards, and there would be no consumption of oxygen as with gas. The outfit for the operating-rooms includes a series of movable reflectors, which enables operations to be performed as well by night as they are by day.

---

At the quarterly court of governors of Addenbrooke's it was stated that the tender of Messrs. Redding and Son for the erection of a ward for sick nurses had been accepted. The committee hope to report the completion of the building at the Midsummer quarterly court. Dr. Mann's festival choir propose to go on giving entertainments in aid of the hospital till they have earned enough to make a substantial and lasting gift to the institution.

---

Princess Louise, accompanied by the Marquis of Lorne, paid a visit last week to the gallery of the Camden School of Art, and opened a loan exhibition of over 200 pictures, in aid of the building fund of the Great Northern Central Hospital, Holloway. The Princess inspected some of the pictures, under the guidance of Mr. Carl Haag. Colonel Milman read an address of welcome to her, which also thanked her for her loan of pictures to the exhibition. The Princess declared the exhibition open. On the motion of the Bishop of Bedford, a vote of thanks was passed to the Princess, which Lord Lorne acknowledged.

---

The subject of leprosy is still very prominently before the public, partly owing to the Prince of Wales's speech at the dinner last week, and partly by the departure of Miss Amy C. Fowler to Molokai. Miss Fowler is the daughter of the Rev. F. W. Fowler, chaplain to Bath Workhouse, but she is a Catholic, and is known in religion as Sister Rose Gertrude. She will be the matron of Father Damien's hospital at Kalawoa, and will try and carry out the work of the heroic priest. Probably Father Damien never dreamed of the vast benefits which would accrue to lepers throughout the world through his devotion and death in their cause. There seems reason to hope that this dread and awful disease will in time perish off the face of the earth, and the first step towards securing this great consummation was taken by Father Damien. Many will watch with earnest interest the career of Sister Rose Gertrude. She should reach Molokai about the middle of February.

The Sanitary Institute issues its second report, which is most satisfactory. During last year three examinations were held, and two courses of lectures were given. Since these examinations were first established 26 examinations have been held, and 718 candidates have been examined, of whom 62 have passed the examination for local surveyors, and 385 that for inspectors of nuisances. The Worcester Congress of September last was also a great success. A sessional meeting for discussion will be held on Feb. 12th ; a course of lectures for sanitary officers will commence on Feb. 18th; and a course of lectures for ladies on Hygiene will probably commence on Feb. 24th.

---

Mr. Armstrong writes to the *Liverpool Post* bringing the old charge against hospitals that they help the survival of the unfittest, and foster the perpetuation of disease. Charity had better be applied to the prevention than to the cure of disease, according to Mr. Armstrong. But is not the higher aim to try and prevent disease, and when that is not possible to try and cure it? Take all preventive measures by all means, but the disease once being there is the poor victim to be cut off from all help and pity? We presume Mr. Armstrong does not suffer from cancer, phthisis, or any other weary and hopeless illness. We presume that if he did he would fly to the nearest physician for relief. It is so easy to generalise, but "Thanks to the human heart by which we live," when special cases come forward the fount of pity will not be stayed.

---

Among those present at the meeting of the Hospitals Association on January 15th, at Westminster Hospital, were : Mr. Henry C. Burdett, in the chair ; Sir William Moore, K.C.I.E., Mr. H. Bonham-Carter (secretary of the Nightingale Fund), Miss Gordon (matron, Charing Cross Hospital), Miss Glossop (member of the Richmond Board of Guardians), Miss A. Griffiths (matron, Lambeth Infirmary), Miss K. Hughes (matron, Grosvenor Hospital for Women and Children), Miss Meadows (matron of the Home and Infirmary for Sick Children, Lower Sydenham), Mr. P. Michelli (secretary, Seamen's Hospital, Greenwich), Miss Honnor Morten, Miss R. Paget (travelling inspector of the Queen's Jubilee Nursing Institute), Mrs. E. E. Perry (matron, Whitechapel Infirmary), Miss Pyne (matron, Westminster Hospital), Mr. Sidney M. Quennell (secretary, Westminster Hospital), the Matron of St. George's-in-the-East Infirmary, Mr. T. Ryan (secretary, St. Mary's Hospital), Dr. J. C. Steele (medical superintendent of Guy's Hospital), and Miss Wilson (secretary of the Workhouse Infirmary Nursing Association).

---

Hospital balls seem to be liable to pros as well as cons. We hear from Birmingham that the authorities of the Queen's Hospital are decided to have shown "a certain amount of neglect" in the case of Joseph Rowlett, who was carried to the hospital during the annual ball, and was left part of the evening without a trained nurse in charge. Then we hear from one of the Asylums Board hospitals that the matron refused to let her nurses dance in the children's ward, upon which the medical officer was appealed to, and overthrew the matron's decision, and dancing was kept up till nearly eleven o'clock. It ought not to be difficult to arrange for this harmless recreation for half the staff at a time, and to so carry it out that the patients suffer no neglect. We know many excellent hospitals where there is an annual dance, and no evil results follow ; but if there are any difficulties at all to be met, the decision should be in the negative. Let the public have no handle for the scandal they are only too ready to raise concerning hospitals. Of course, the Asylums Board cannot hope to secure good matrons if they do not uphold their authority, but this is a subject we must refer to next week at length.

